# Learning of Spatial Properties of a Large-Scale Virtual City With an Interactive Map

**DOI:** 10.3389/fnhum.2019.00240

**Published:** 2019-07-10

**Authors:** Sabine U. König, Viviane Clay, Debora Nolte, Laura Duesberg, Nicolas Kuske, Peter König

**Affiliations:** ^1^Institute of Cognitive Science, Osnabrück University, Osnabrück, Germany; ^2^Department of Neurophysiology and Pathophysiology, University Medical Center Hamburg-Eppendorf, Hamburg, Germany

**Keywords:** spatial memory, navigation, virtual reality, interactive city map, spatial cognition

## Abstract

To become acquainted with large-scale environments such as cities people combine direct experience and indirect sources such as maps. To ascertain which type of spatial knowledge is acquired by which source is difficult to evaluate. Using virtual reality enables the possibility to investigate whether knowledge is learned by direct experience or the use of a map differentially. Therefore, we designed a large virtual city, comprised of over 200 houses, and evaluated spatial knowledge acquisition after city exploration with an interactive map following one and three 30-min exploration sessions. We tested subjects’ knowledge of the orientation of houses facing directions toward cardinal north, of orientations of houses facing directions relative to each other and pointing from one house to another. Our results revealed that increased familiarity after extended exploration with the map improved task accuracy. Further, it revealed task differences, caused mainly by a better accuracy in the relative orientation task than the pointing task. Time for cognitive reasoning improved overall task accuracy. Learning with our VR city map revealed an absence of distance effect, an alignment effect of tested house orientation toward map north and an angular difference effect between tested stimuli. Self-reported knowledge of cardinal directions learned in the real environment was positively correlated with task accuracy testing houses orientations toward cardinal north. Overall, our results suggest that participants learned spatial information that is directly available in the interactive map, while a spatial task that needed integration of learned knowledge stayed at lower accuracy levels.

## Introduction

Spatial navigation in large-scale environments is an essential ability for many aspects of everyday life. Learning the spatial layout and spatial relations of your surroundings is based on different sources. People acquire spatial knowledge through direct experience, but they also use indirect sources like maps to acquire spatial knowledge (Shelton and McNamara, 2004; Meilinger et al., 2013). Those different sources provide different spatial information, e.g., about locomotion of the body, perspective, and scale of the environment, metric information, and precision of the presented spatial knowledge, thus also leading to differences in spatial knowledge acquisition and spatial memory (Richardson et al., 1999; Montello et al., 2004). Investigating learning of everyday navigation gives insights into acquired spatial knowledge by a combination of different sources.

The classic framework of spatial knowledge acquisition of large-scale environments by Siegel and White (1975) introduced three different types of spatial knowledge. With direct experience of an environment, people acquire knowledge of salient objects in the environment, called landmarks. This landmark knowledge is supposed to have no metric information. Through traveling connecting routes between landmarks, route knowledge evolves. Route knowledge can be seen as a sequence of acquired knowledge about the space between landmarks derived from direct navigation experience of the navigator. Integration of route knowledge leads to a map-like representation of metric spatial relationships, so-called survey knowledge. Survey knowledge relates spatial knowledge to a fixed coordinate system, e.g., compass bearings, which allows taking shortcuts not traveled before and straight line pointing. The classical view of spatial knowledge acquisition (Siegel and White, 1975) suggested that spatial learning evolves in a stepwise sequence of landmark, route, and survey knowledge with increased familiarity of the environment. More recently, Montello (1998) introduced a new framework, which especially suggests that after only one exposure to the environment acquired spatial knowledge contains already metric information about distances and directions. This is supported by empirical studies showing that early spatial learning directly includes knowledge about metric properties of the environment such as distances and angles between locations (Klatzky et al., 1990; Montello and Pick, 1993). That framework also proposes that more familiarity with the environment increases the quantity and accuracy of the spatial knowledge but the change is more continuous and quantitative than qualitative. With the increasing familiarity of a location, spatial knowledge becomes more precise and confident (Montello, 1998; Ishikawa and Montello, 2006; Iachini et al., 2009; Nori and Piccardi, 2010; Piccardi et al., 2011). In contrast to the classic framework, studies have shown that also survey knowledge can be acquired by minimal experience in the environment (Klatzky et al., 1990; Loomis et al., 1993). But there are substantial individual differences in spatial knowledge acquisition. These individual differences are especially marked in acquiring survey knowledge that requires integration of spatial knowledge (Montello, 1998; Ishikawa and Montello, 2006; Wiener et al., 2009). However, what type of spatial knowledge is acquired is also influenced by the source of learning. Using active navigation is supposed to especially improve route knowledge (Thorndyke and Hayes-Roth, 1982; Taylor et al., 1999; Shelton and McNamara, 2004; Meilinger et al., 2013), whereas the acquisition of survey knowledge of a large-scale complex environment is more supported by the use of a map (Thorndyke and Hayes-Roth, 1982; Taylor et al., 1999; Montello et al., 2004; Meilinger et al., 2013). Nevertheless, the kind of spatial knowledge that is used in a specific situation depends on the particular task and likely combines multiple types of knowledge (Meilinger et al., 2013).

The source of spatial learning also influences the kind of reference frame in which spatial features are learned and memorized. The most crucial distinction of spatial reference frames is drawn between egocentric and allocentric reference frames (Klatzky, 1998; Mou et al., 2004; Burgess, 2006). An egocentric reference frame relates the environment to the physical body of the navigator (Klatzky, 1998). Meilinger et al. (2013) discovered that route knowledge, which is supported by direct experience in the environment, improved when participants responded in a route recall task from a pedestrian perspective. This suggests that route knowledge is coded with respect to the physical body in an egocentric reference frame (Siegel and White, 1975; Shelton and McNamara, 2001). While moving in the environment also spatial updating, which is caused by changes in somatosensory information, is coded in an egocentric reference frame (Wang and Brockmole, 2003; Riecke et al., 2007). Instead, spatial knowledge coded in an allocentric reference frame is based on allocentric bearing and distance independent of the physical body of the observer (Klatzky, 1998). Thus, getting acquainted with an environment by a map, which provides information on cardinal directions and spatial orientation, presumably supports spatial knowledge coded in an allocentric reference frame. This suggests that survey knowledge, which evolves more accurately when learned from a map, is also coded in an allocentric reference frame (Siegel and White, 1975; Thorndyke and Hayes-Roth, 1982; Richardson et al., 1999; Montello et al., 2004). Even though spatial knowledge is stored in distinct reference frames, they are supposed to develop together (Nardini et al., 2006) and to be combined for active navigation (Burgess, 2006; Ishikawa and Montello, 2006; Gramann, 2013; Meilinger et al., 2013). Nevertheless, it can be suggested that direct experience in the environment supports route knowledge to be preferentially coded in an egocentric reference frame, whereas spatial learning with a map preferentially leads to survey knowledge that is coded in an allocentric reference frame.

Additionally, to spatial knowledge coded with respect to reference frames, the specificity of remembered spatial knowledge with respect to orientation is an essential question in spatial navigation research (Montello et al., 2004). Several studies report systematic differences in task accuracy or response times as a function of orientations while learning or testing spatial knowledge (e.g., Presson and Hazelrigg, 1984; Sholl and Nolin, 1997; Waller et al., 2002; Shelton and McNamara, 2004; Sholl et al., 2006; Frankenstein et al., 2012; Burte and Hegarty, 2014). Here, orientation specificity is visible in an alignment effect that is characterized by high performance when the learning and testing orientations are aligned and a decrease of performance coincides with an increase of angular difference between orientations. This also suggests that metric information is directly acquired during spatial learning leading to differential behavior. The alignment can be based on the physical body (physical facing direction) relative to directions toward objects or locations in the environment, thus, being based upon an egocentric reference. Here, the accuracy in pointing tasks was best when the imagined recall direction was aligned with the egocentric direction from which the environment was learned (Shelton and Mcnamara, 1997; Shelton and McNamara, 2001; McNamara, 2003; McNamara et al., 2008; Meilinger et al., 2013; Burte and Hegarty, 2014) or when the imagined orientation was aligned with the physical orientation of the participant (Waller et al., 2002; Burte and Hegarty, 2014). But the alignment can also be based upon an allocentric reference. Here pointing accuracy in a large-scale environment was best when cognitive maps or tested objects orientations were aligned relative to environmental features (McNamara, 2003; McNamara et al., 2003; Brunyé et al., 2015) or fixed directions like cardinal directions (Frankenstein et al., 2012; Brunyé et al., 2015). In an allocentric-heading recall task, investigators found an alignment effect between the physical facing direction of a participant in a known environment with the orientation of a photograph (Sholl et al., 2006; Sholl, 2008). Here, alignment between physical facing direction and facing direction of a photograph (the direction from which the photograph was taken) reduced the decision time. Judging the facing direction of photographs was correlated with self-reported sense of direction (SOD) (Sholl et al., 2006) revealing large individual differences. In a later study, Burte and Hegarty (2014) compared in a relative heading task two allocentric headings of photographs (orienting photographs and target photographs). Here, the allocentric heading, defined as the angle between the objects axis of orientation and a reference orientation (Klatzky, 1998) of the orienting photograph, did not match the physical heading of the participants. In this study, they found a significant alignment effect of the target photographs’ heading toward environmental structures onto accuracy and decision latency, but they failed to reproduce an alignment effect between orienting- and target photographs’ heading. As the main factor that determines orientation specific spatial knowledge Presson and Hazelrigg (1984) identified the source for spatial knowledge acquisition. Spatial knowledge that was learned by direct experience, either walking or viewing, revealed no alignment effect and thus an orientation free spatial knowledge whereas learning with an indirect source like a map led to orientation specific knowledge visible in an alignment effect. Overall, when spatial layouts of large-scale environments were learned with a map, researchers found a reliable alignment effect suggesting that an orientation specificity was acquired with a preferred orientation of the map (Evans and Pezdek, 1980; Levine et al., 1984; MacEachren, 1992; Montello et al., 2004; Shelton and McNamara, 2004).

For the learning of large complex environments such as cities, in western countries cartographical 2-dimensional (2D), north-up maps from a bird’s eye view are most often used. Those maps depict the real world in downscale while keeping the proportions intact. Thus, distances and locations, as well as spatial orientation, resemble the real world situation. In modern times, technology on digital devices allows switching from cartographical 2D maps to 3-dimensional (3D) interactive maps like Google Street View. In interactive city maps, observers change their perspective from a bird’s eye view to a pedestrian view, mimicking an embodied perspective, while navigating. A recent report compared the performance in orientation tasks after learning with 2D and 3D city maps (Oulasvirta et al., 2009). They found that learning from 2D maps yielded better performance than from 3D maps, even in participants who were used to 3D navigation through the gaming experience. Overall, 3D maps supply a pedestrian perspective of the environment, whereas 2D maps provide the observer with the information of metric properties of large-scale environments from a bird’s eye view. Thus, 2D and 3D maps provide different spatial information supporting different aspects of spatial knowledge acquisition causing additional cognitive effort when switching from one to the other map.

Numerous studies investigated spatial knowledge acquisition with direct experience and indirect sources like maps in small and larger scale environments. However, it is challenging to get differential insights into spatial knowledge that was acquired and is used naturally in everyday navigation in a large-scale environment like ones hometown. A previous study (König et al., 2017) investigated allocentric spatial knowledge retrieval after everyday navigation in the hometown of Osnabrück, Germany. They used photographs of houses and streets as stimuli and tested spatial knowledge of the orientation of houses and streets toward cardinal north, the relative orientation of two houses and two streets, respectively, and relative location of two houses in a pointing task. Investigating spontaneous knowledge retrieval, houses were best remembered in the house-to-house relations, whereas streets were preferentially coded in relation to cardinal directions. Time for cognitive reasoning also improved the knowledge of the cardinal orientation of houses. This study faced the problem that measuring spatial abilities obtained by everyday navigation does not allow separating different sources in the acquisition of spatial knowledge. Furthermore, living in a city people are more familiar with some parts and do not know other parts of the city resulting in substantial differences of familiarity. As the actual spatial acquisition is in these situations only measured after it was gained, familiarity can only be subjectively investigated (König et al., 2017). Progress of technology in virtual reality (VR) renders it possible to design large-scale more naturalistic environments in VR. These VR environments support the investigation of spatial navigation and spatial learning with respect to embodied navigation and the possibility to investigate spatial learning under controlled conditions. Thus, investigations of learning by direct experience in VR and acquiring knowledge about the VR environment with a map can be performed separately.

In the presented study, we, therefore, explored knowledge acquisition of spatial properties in a large virtual city with controlled exploration. We report here only the exploration with an interactive city map. The results after direct experience in VR are reported in a separate study. For this investigation, we built a large virtual city named Seahaven, which would cover 500 m × 431 m in real-world measurements and contains 213 houses. In the present study, all participants freely explored the virtual city using an interactive city map for one or three 30 min sessions resulting in 30 or 90 min of exploration. The map displayed the city layout in 2D, north up, bird’s eye perspective with the addition of an interactive feature. This feature enabled to view the front-on screenshots of houses when the participant clicked on the respective house on the map. Our research question was whether participants using the VR city map would acquire spatial knowledge about orientation of houses facing directions toward the north cardinal direction (heading of a house) (absolute orientation task), orientation of houses facing directions in relation to the reference orientation of a prime house facing direction (relative heading of houses) (relative orientation task) and survey knowledge that allowed straight line pointing from one house to another (pointing task). We tested all tasks with a spontaneous response (within 3 s) and time for cognitive reasoning (infinite time to respond). This choice of response times was supported by Dual-Process Theories that distinguish between rapid, automatic, and associative so called “System 1” cognitive processes and slow, analytic, and deductive so called “System 2” cognitive processes (Evans, 1984, 2008; Kahneman et al., 2002; Kahneman, 2011). These works do not make precise quantitative statements on how fast is fast. However, values of 3 s and 5 s reappear as boundaries based on different reasoning (Finuncane et al., 2000; Kahneman, 2011). Further, in line with work on the speed accuracy tradeoff (Heitz, 2014), 3 s appears to be a reasonable choice of response time for our investigations. To investigate our research questions, we adjusted and performed the navigation tasks, which were used in a previous study (König et al., 2017). Based on the results of this previous study (König et al., 2017) we hypothesized that with infinite response time for cognitive reasoning, participants would perform more accurately than when spontaneous decisions within 3 s were required. As they are provided with the information of cardinal directions directly from the map without the need for further cognitive reasoning, we hypothesized that participants would perform more accurately at determining the absolute orientation of a single house toward the north than the relative orientation of two houses or the straight line pointing between houses. Following earlier reports investigating effects of increased familiarity (e.g., Montello, 1998), we expected to find improved task accuracy with increased exploration time. Additionally, we assumed that the accuracy would improve the more often a house was looked at during exploration. Testing the angular difference between task choices, we expected to find an improved accuracy with larger angular differences between stimuli choices, whereas increased distance between tested houses would yield no accuracy difference in line with studies investigating spatial learning with a map (Loomis et al., 1993; Meilinger et al., 2015). We further explored whether spatial orientation strategies based on egocentric or allocentric reference frames that are used in everyday navigation have an impact onto learning of spatial properties tested in our tasks after exploring a virtual city with an interactive map. For this purpose, we performed the self-report measure “Fragebogen Räumlicher Strategien” (FRS, translated as the German Questionnaire of Spatial Strategies) that captures egocentric and allocentric spatial orientation strategies (Münzer and Hölscher, 2011; Münzer et al., 2016b). Overall, we investigated controlled spatial learning with an interactive city map of a virtual city to get more insight into the differential learning of spatial properties.

## Materials and Methods

### Participants

Seventy-seven young, healthy adults (40 females, mean age of 24.0 years, SD = 3.9) took part in our study using an interactive map of a virtual city for spatial learning. Seven participants had to be excluded because of technical problems during the first measurement. All of the remaining 70 participants performed one 30-min exploration session with the interactive map and performed the spatial tasks once. This slightly surpasses the target of 66 valid subjects as determined by (G^∗^Power 3.1, effect size = 0.25, alpha = 0.05) analysis. 22 of these participants explored the city with the map and performed all spatial tests repeatedly two additional times on three separate days within ten days resulting in a total exploration time of 90 min (11 females, mean age of 23.8 years, SD = 3.1). Before the start of the experiment, participants were informed about the purpose and the procedures of the experiment, and gave written informed consent. At the end of the experiment, they performed the FRS questionnaire [“Fragebogen Räumlicher Strategien,” translated “Questionnaire of Spatial Strategies” (Münzer and Hölscher, 2011)]. Each participant was either reimbursed with nine Euros per hour or earned an equivalent amount of “participant hours,” which are a requirement in most students’ study programs. Overall, the experiment took about 2 h per session. The study was approved by the ethics committee of the Osnabrück University in accordance with the ethical standards of the Institutional and National Research Committees.

### Experiment Procedure

Our experiment contained four major phases, which are described in detail below (Table 1). The first was the introductory phase, which lasted approximately 30 min. Participants were first informed about the experiment and gave written informed consent. Then, they performed a response training and received spatial task instructions and training with example trials of all spatial tasks and time conditions. Next, participants were introduced into the interactive map of the virtual city and how to use it to explore the city. After this followed the exploration phase, in which participants freely explored the city solely with the interactive city map for 30 min. The experiment was followed with the test phase lasting for approximately 45 min, where participants were tested on three different spatial tasks (absolute orientation-, relative orientation-, and pointing task) in two time conditions (3 s and infinite response time) (Table 1). After the spatial tasks, participants filled out a questionnaire on spatial strategies (FRS questionnaire), which concluded the experiment. The spatial tasks were in close analogy to a previous study performed in our laboratory (König et al., 2017). Other groups of participants explored the city in VR with or without the feelSpace belt, a device supplying information about magnetic north (Kaspar et al., 2014; König et al., 2016). However, these data are not covered in the present report. Here, we report the results of participants, who were used an interactive city map of Seahaven.

**Table 1:** Experiment procedure.

Single steps in the experiment		Phases of the experiment
1	Subject information, written informed consent	Introductory phase (30 min)
2	Response training	
3	Spatial tasks instructions	
4	Tasks training with example trials of all tasks in both time conditions	
5	Introduction of map exploration	
6	Free exploration with the interactive city map	Exploration phase (30 min)
7	Spatial tasks (absolute orientation-, relative orientation-, pointing tasks) in two time conditions (3 s to respond, infinite time to respond)	Test phase (45 min)
8	FRS Questionnaire	Questionnaire phase (5 min)

### Response Training

To familiarize participants with the 3 s response and how to understand the directional arrow on the screen and what the required behavioral response was, each participant performed a response training. In this training, one arrow surrounded by an ellipsoid appeared on the upper screen, and another appeared on the lower screen, each pointing in different directions. The participants had to compare the two arrows and then select within 3 s the arrow that pointed more straight upward on the screen, which indicated north on the map. To select the arrow on the upper screen, they had to press the “up” button and to select the arrow on the lower screen they had to press the “down” button. On each trial, they got feedback as to whether they decided correctly (green frame), incorrectly (red frame), or failed to respond in time (blue frame). The response training was finished, when the participants responded correctly without misses in 48 of 50 trials (>95%) (König et al., 2017). This response training ensured that participants were well acquainted with the response mechanism of the two alternative forced choice responses that our spatial tasks used.

### Spatial Tasks Instructions and Tasks Training

Each participant received task instructions and task training before the start of the free exploration time of Seahaven. It is known that during spatial learning it is necessary to pay attention to the environment or the map to gain spatial knowledge (Montello, 1998). A separate pilot study revealed that during their free exploration of the city participants sometimes focused more on aspects, like detailed house design that would not support spatial learning. Therefore, we introduced the spatial tasks to support intentional learning. Please note that none of the subjects of the pilot study is included in the main study and that no subject of the main study was excluded for reasons of their spatial exploration behavior. The instructions were given by written and verbal explanations using photographs of houses in the city of Osnabrück that were used as stimuli in a previous study (König et al., 2017). Participants then performed a pre-task training with one example of all spatial tasks each in both time conditions to gain a better insight into the actual task requirements. This pre-task training utilized photographs of houses of the city Osnabrück to avoid the possible transfer of training effects onto the stimulus set of Seahaven. Except for the stimuli, the pre-tasks resembled exactly the spatial tasks design (see in the experimental design below). We performed the pre-task training to familiarize participants with the spatial knowledge that was tested in the spatial tasks.

### The Virtual City “Seahaven”

**FIGURE 1:**
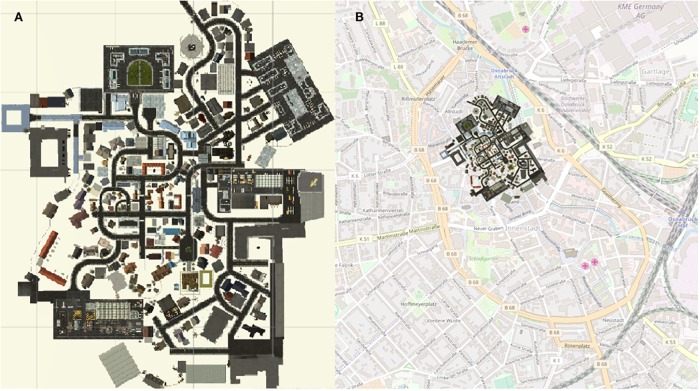
**(A)** City map of Seahaven depicting all houses that can be clicked on, to display a screenshot of the front-on view of the respective house. **(B)** City map of the virtual city Seahaven overlaid onto the city map of central Osnabrück.

To be able to investigate the spatial exploration and acquisition of spatial knowledge of an unknown environment, we designed a virtual city, called Seahaven. The name Seahaven is derived from the name of the town in Peter Weir’s 1998 film “The Truman Show,” in which the main character Truman Burbank is also living in a small town with no possibility to leave while every moment is watched and analyzed by many people, albeit in the form of a reality TV show. Our virtual city Seahaven was built in the Unity^®^ game engine. Seahaven covers 500 × 431 Unity^®^ units. 1 Unity^®^ unit was designed to resemble 1 m in real-world measure. Thus, Seahaven covers 0.216 km^2^ in real-world measures (Figure 1). Seahaven contains 213 houses in diverse styles. Considering bins of 30° size, the number of houses sharing a particular orientation toward North is approximately equally distributed around the full circle. Compared to many virtual environments used for spatial navigation research, Seahaven is large and complex. Compared to the real city of Osnabrück, the size of Seahaven is approximately one-fifth of the inner city surrounded by the “Wall” (Figure 1B). This is a size that can be well explored within half an hour. For the following spatial navigation tasks, Seahaven does not contain tall landmarks or specific city districts, and the street system does not follow an ordered grid (i.e., “Manhattan style”). All files required for the conduction of experiments are available on the GitHub account of one of the authors (V. Kakerbeck, married VC) (Clay et al., 2019).

### Introduction of Map Exploration With an Interactive City Map of Seahaven

**FIGURE 2:**
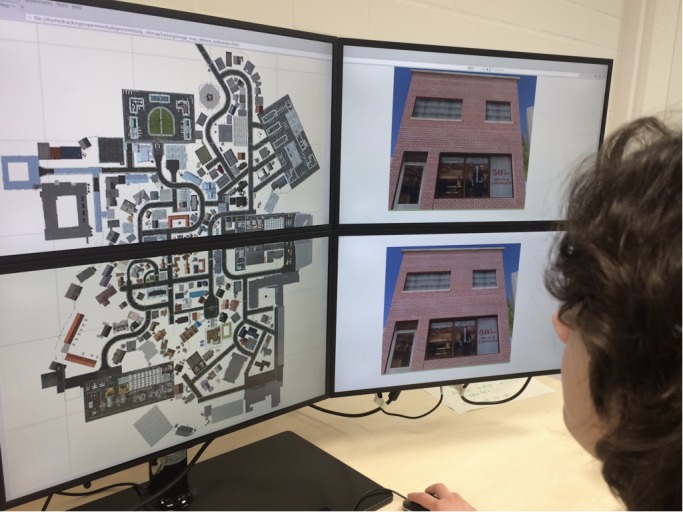
Experimental set-up of the interactive city map of Seahaven. Left, birds-eye view, north up city map. Right, the screenshot of the clicked house appears on the two-stacked screens on the right. Written informed consent was obtained from the individual for the publication of this image.

To get to know the city of Seahaven, participants freely explored it in one or three repeated sessions of 30 min with a two-dimensional north-up interactive city map. This resulted in 30 or 90 min exploration time. Participants sat approximately 60 cm from a six-screen monitor setup in a 2 × 3 screen arrangement. During the exploration, the map was presented on the two central screens, one above the other (Figure 2, left). This map resembled a traditional city map with a north-up orientation and a bird’s-eye view. It was implemented using HTML, jQuery, and CSS. The map provided the participants with task-relevant spatial information about cardinal directions and the location, orientation, and relation of houses. By adding an interactive component, participants were also provided with the screenshots of front-on views of 193 houses in Seahaven that were used as spatial task stimuli. To view the houses’ screenshots, participants moved with a mouse over the map. When hovering over a house, a red dot appeared on one side of the respective house. The red dot indicated the side of the house that was displayed in the screenshot. By clicking on this house, the screenshot was displayed twice on the two right screens of the monitor (the same image above each other) (Figure 2, right). How often houses were clicked on was recorded and later used to determine participants’ familiarity with the stimuli and which houses were looked at to investigate the part of Seahaven that participants visited. The two screens on the left side were not used during exploration or testing. During the city exploration participants got feedback on how much time had passed after 15 and 25 min. They had a short break of 2–5 min between the exploration and testing of spatial tasks. In summary, the interactive map provided participants with a city map of Seahaven and the front on views of the majority of houses of Seahaven.

### Stimuli

**FIGURE 3:**
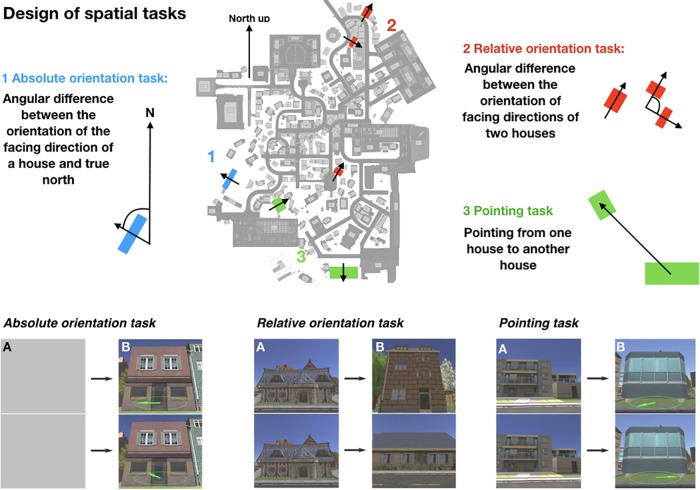
Design of the spatial tasks depicting the schemata of the absolute orientation task in blue (1), the schemata of the relative orientation task in red (2), and the schemata of the pointing task in green (3). On the lower part, example trials of the spatial tasks are shown. On the left side a trial of the absolute orientation task, in the middle a trial of the relative orientation task, and on the right a trial of the pointing task is depicted. Task stimuli used screenshots of houses in the virtual city. First, a prime stimulus **(A)** is shown for 5 s in the relative and in the pointing task, which is substituted by a gray screen in the absolute orientation task to fit the experimental condition in the other tasks. It is followed by a target stimulus **(B)** shown until button press max in 3 s or with infinite time on two monitors, one above the other. In the absolute orientation task, participants had to choose the arrow depicted on the stimuli that correctly pointed to map north. In the relative orientation task, they had to select the target house that had the same orientation as the prime house. In the pointing task, participants had to choose the target stimulus on which the arrow pointed correctly to the prime house. For more details see section “Materials and Methods.”

Our stimuli were front-on screenshots of 193 houses of the overall 213 houses in Seahaven (examples are shown in Figure 3). In our tasks, we compared the orientations of the facing directions of the houses. The facing direction of a house was the facing direction of the photographer when taking the screenshot of the respective house. The heading of a house is the angle between the facing direction of the house and a reference direction, e.g., cardinal north. The photographer took the screenshots in the virtual environment from a pedestrian viewpoint that would resemble approximately 5-m distance to the corresponding house from a position on a street or other walkable paths in the VR city. For some houses, this was not possible so they were excluded as stimuli. Furthermore, a few houses looked too similar to each other and therefore had to be excluded as well. All screenshots were scaled to 1920 × 1080 pixels so that each screenshot was presented in full screen on one of the six monitors. For the prime stimuli in the relative orientation and pointing tasks, we used the screenshots of 18 houses that were most often viewed in a VR pilot study (although the present subjects experienced the city exclusively by the interactive map). These prime houses were distributed equally over the possible angles differing from map cardinal north in steps of 30° and were spread well across the city. They were used twice in the tasks consisting of 36 trials. The stimuli that were used as target stimuli were only used once and were not shown repeatedly. In the spatial tasks, we used a subset of all screenshots that were used in the interactive city map training.

### Spatial Tasks

The design of the spatial tasks was adapted from a previous study by König et al. (2017). We randomized the blocks of all task and time conditions that are described in the following to prevent a systematic bias by learning effects over time.

#### Absolute Orientation Task

In the absolute orientation task, we measured participants’ knowledge about the orientation of single houses facing direction in relation to the cardinal north direction (heading). Therefore, we designed a two-alternative forced choice (2AFC) task with screenshots of front-on views of houses of the virtual city Seahaven as stimuli (Figure 3, absolute orientation task in blue). The facing direction of a house depicted the photographers viewing direction while taking the screenshot. We presented the same house stimulus twice on the stacked middle screens of our 6-screen monitor (Figure 3B). An arrow within an ellipsoid depicting a compass was overlaid on each of the two stimuli. One of the arrows pointed correctly to the northern direction from the facing direction of the house front, whereas the other arrow pointed randomly in a direction that diverged from the north by some amount in steps of 30°. As we expected larger angular differences to be easier to learn, we randomized the angular difference in the absolute orientation task between the correct arrow and the wrong arrow. Participants had to choose, which arrow pointed correctly toward north by pressing either the “up” (upper screen) or “down” (lower screen) button on the response box. We used two different time conditions: 3 s and infinite response time to investigate the influence of spontaneous decisions compared to decisions with time for cognitive reasoning, respectively. One trial consisted of a gray screen shown for 5 s on both middle screens, which was followed by the stimulus set (Figure 3, absolute orientation task A and B). After the press of a response button, either within 3 s or with infinite time for the decision, again the two central gray screens appeared followed by another stimulus set. Both time conditions contained 36 trials. House stimuli were only used once. The gray screens were interposed between the experimental trials to have the same time course as in the two other tasks (see the absolute orientation and pointing task). In case the subject did not respond within the time limit, the trial was counted as incorrect. This was done as well in the other tasks. The two time conditions were blocked and introduced by written instructions on the screens.

#### Relative Orientation Task

In the relative orientation task, we measured the knowledge to estimate the orientation of houses facing direction relative to each other (Figure 3, relative orientation task in red). Here, the orientation of the facing directions of two target houses had to be judged in relation to a reference orientation given by the facing direction of a prime house (relative heading). We, therefore, designed a 2AFC task with front-on views of houses of the virtual city Seahaven as stimuli again with either 3 s or infinite time to respond. In the task design of the relative orientation task, each trial depicted a stimulus set consisting of a fixed triplet of images: one priming image, whose orientation was the reference orientation to which the orientations of two target images had to be compared (Figure 3, relative orientation task A and B). The orientations of the target houses facing directions deviated from each other in steps of 30°. We randomized this angular difference between the two target houses. In each trial, the priming image was first shown on both middle screens for 5 s. After the priming stimulus was turned off, the two target stimuli appeared, one on the upper screen and the other on the lower screen. For a few houses, it was not possible to perfectly align the prime and correct target stimuli to 0°. Therefore, the task of the participants was to select the target image that was more closely aligned (± 5°) with the orientation of the priming image by pressing either the “up” or “down” button on a response box, respectively. Again, this sequence was repeated with 36 stimulus sets in each time condition. Eighteen preselected prime stimuli were shown twice, whereas all target stimuli were only shown once. The time conditions were blocked and introduced by written instructions on the screens.

#### Pointing Task

In the pointing task, an established paradigm in spatial navigation studies, we investigated the knowledge of the spatial relation between the locations of two houses in Seahaven by straight line pointing (Figure 3, pointing task in green). We used the same stimulus material of houses as before. Here, we defined pairs of houses with a priming stimulus and a target stimulus. First, a priming image appeared on two stacked screens for 5 s (Figure 3A, pointing task), which was followed by a target stimulus, the same on both screens. One target stimulus was overlaid with an arrow, which correctly pointed into the direction from the target to the priming image location. On the second stimulus of the target house, the arrow was pointing in a random direction differing with varying degrees in steps of 30° from the correct direction (Figure 3B, pointing task). We randomized the angular difference in the pointing task between the correct and wrong arrow of the target house pointing to the prime house. The pointing task was again performed in the two time conditions with 3 s to respond or infinite time to respond. In a 2AFC task, participants had to choose the target stimulus on which the arrow pointed correctly from the target house toward the priming house by pressing either the “up” or “down” key. The pointing task consisted again of 36 trials in each time condition, which were blocked and introduced by written instructions on the screen. Eighteen preselected prime stimuli were shown twice, whereas all target stimuli were only shown once.

### FRS Questionnaire

At the end of the measurements, participants filled in the “Fragebogen Räumlicher Strategien” (FRS) questionnaire, translated “Questionnaire of Spatial Strategies” (Münzer and Hölscher, 2011). The FRS questionnaire imposes self-report measures for spatial orientation strategies learned in real environments. It captures three different scales. The “global-egocentric scale” evaluates global orientation abilities and egocentric abilities based on knowledge of routes and directions. The “survey scale” assesses an allocentric strategy for mental map formation. The “cardinal directions scale” evaluates knowledge of cardinal directions. Each scale consists of Likert items with a score ranging from 1 (“I disagree strongly.”) to 7 (“I agree strongly.”). The FRS questionnaire evaluates with the first factor strategies based on an egocentric reference, whereas the second factor captures an allocentric survey strategy and the third an allocentric strategy using cardinal directions (Münzer and Hölscher, 2011; Münzer et al., 2016a,b). Thus, the FRS questionnaire enables us, to get insight into the preferred use of egocentric or allocentric spatial strategies.

## Results

Here, we report the results of participants using the interactive city map to explore our virtual city. A total of 70 participants were investigated after one exploration session of 30 min. Out of these, 22 participants conducted three repeated sessions resulting in an exploration time with the map of 90 min. All reported tests use a within-subject design.

### Results of Exploration Behavior With the Interactive City Map

**FIGURE 4:**
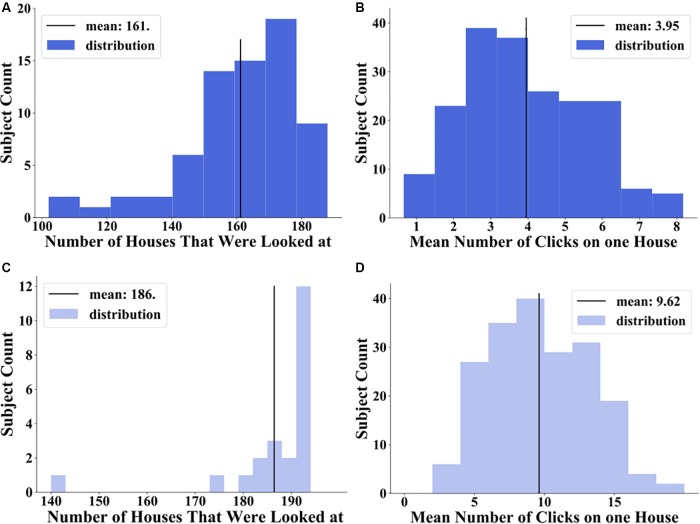
Distribution of number of houses that were looked at: **(A)** after 30 min exploration in middle blue and **(C)** after 90 min exploration in light blue and distribution of mean number of clicks on one house over all participants: **(B)** after 30 min exploration in middle blue and **(D)** after 90 min exploration in light blue.

To evaluate the exploration of the city with the interactive city map, we determined how often houses were clicked on and how many houses the participants viewed. In the first exploration session, the number of houses that were looked at by each participant ranged from 102 (52.85%) to 188 houses (97.41%) out of 193 possible houses, resulting in a grand average of 161 houses (83.42%) (Figure 4A). The mean number of clicks on a house made by a participant ranged in the first exploration session from 0.67 to 8.14 clicks, resulting in a grand average of 3.95 clicks on a house (Figure 4B). Thus, the half-hour exploration time was sufficient to explore a larger fraction of all houses consistently. Accumulating the results of first, second, and third exploration session, participants had looked at between 131 (68%) and 193 (100%) houses resulting in a grand average of 186 houses (96.37%) that were viewed (Figure 4C). The mean number of clicks on a house made by a participant ranged after the third exploration session from 2.41 to 18.68 clicks, resulting in a grand average of 9.62 clicks on a house (Figure 4D).

**FIGURE 5:**
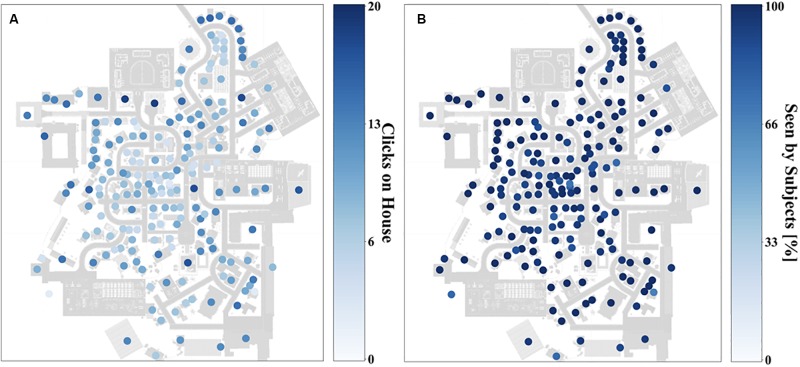
Heat map depicting how often participants clicked on houses **(A)** and how many participants looked at a house **(B)** on a gradient going from deep blue (most often) to white (zero) after 90 min exploration.

The visualization of the spatial distribution on the city map (Figure 5) revealed a good coverage of clicked on and therefore viewed houses over the city map. The majority of participants looked at most houses. Houses on the outer parts of the city were more often clicked on, in contrast to centrally located houses after the first session. Accordingly, the houses on the outer parts of the city were looked at in a higher frequency than the more centrally located houses. After three sessions only a small bias of more frequent clicked on houses in the periphery of the city map remained (Figure 5A) and all of the participants saw most of the houses (Figure 5B). However, we did not find systematically neglected areas of the city. This shows that the spatial exploration with the interactive map covers the whole city with a small bias to more frequently viewed houses on the outskirts of Seahaven.

### Spatial Task Results

In this paper, our main focus was to investigate the dependencies of performance measured in the accuracy of task choices in the 2AFC spatial tasks after 30- and 90-min exploration with an interactive city map. We first focused on a comparison of the different task and time conditions. Here, we compared the accuracy of the tasks after 30 and 90 min to evaluate the influence of familiarity. Next, we considered the clicks on houses as an objective measure for how frequently a house was viewed. Furthermore, we investigated the distance between tested stimuli, the angular difference between tested stimuli, and the alignment toward map north as relevant factors. Additionally, we investigated the influence of subjectively rated abilities of spatial strategies using the FRS questionnaire (Münzer and Hölscher, 2011) on task accuracy.

#### Accuracy in Different Spatial Task and Decision Time Conditions After One and Three Exploration Sessions

We hypothesized that participants using an interactive city map for the exploration of the virtual city perform more accurately with infinite time for a decision and thus for cognitive reasoning than in the spontaneous decision mode with 3 s response time. Furthermore, we assumed that participants exploring the city with a map would perform better in the absolute orientation task than in the relative orientation and pointing task. Additionally, we hypothesized that with increased time for exploration and thus increased familiarity with Seahaven participants’ tasks accuracy would improve.

**FIGURE 6:**
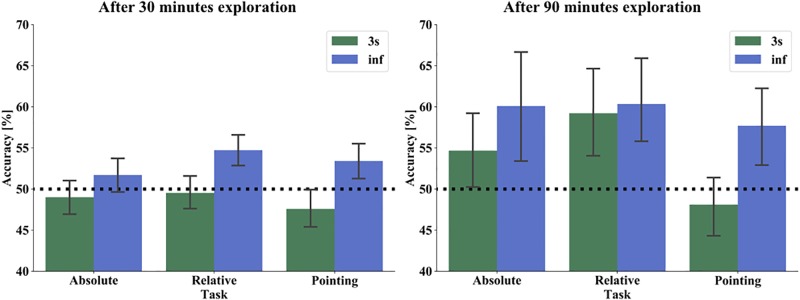
Performance of absolute orientation (left), relative orientation (middle), and pointing task (right) in 3 s (green) and infinite time (blue) condition after 30 min (left) and 90 min (right) exploration. The bars show the mean performance of all subjects in both time response conditions. The black dashed line marks the chance level of 50%. The error bars indicate 95% confidence interval.

Therefore, we calculated the accuracy as the fraction of correct answers per participant for each task and each time condition. We performed a within-subject repeated measure ANOVA with accuracy as the dependent variable and time (3 s, infinite) and task (absolute orientation, relative orientation, and pointing) as repeated factors in a 2 × 3 design (Figure 6). After 30 min of exploration, the accuracy in the tasks were as followed: absolute task/3 s 49.01%, relative tasks/3 s 49.52%, pointing/3 s 47.58%, absolute task/infinite 51.70%, relative task/infinite 54.72%, and pointing/infinite 53.41% (Figure 6). The ANOVA revealed a significant main effect for time (F(1,69) = 24.104, *p* < 0.001, η^2^ = 0.259), but no significant main effect for task (F(2,138) = 1.693, *p* = 0.188) and no significant time × task interaction (F(2,138) = 1.393, *p* = 0.252). After 30 min of exploration, we found as hypothesized a significantly better accuracy with the infinite response time. Our results revealed accuracy in the 3 s condition after 30 min exploration slightly below 50% and significantly lower than in the infinite time condition. Effectively, this is a floor effect and a further investigation of the 3 s condition after one session seemed not to be meaningful. Therefore, we calculated further results for the first exploration session taking into account only the infinite time condition (different from chance with *p* = 0.026). After the first session, we did not find a significant main effect for task as expected.

After 90 min of exploration with the map, the accuracy improved throughout: absolute task/3 s 54.67%, relative tasks/3 s 59.21%, pointing/3 s 48.11%, absolute task/infinite 60.10%, relative task/infinite 60.35%, and pointing/infinite 57.70% (Figure 6). In the within-subject repeated measure ANOVA with the same factors as after one session, we found a significant main effect for time (F(1,21) = 11.963, *p* = 0.0023, η^2^ = 0.363), a main effect for task (F(2,42) = 6.353, *p* = 0.0039, η^2^ = 0.232), but no significant time^∗^task interaction (F(2,42) = 2.370, *p* = 0.1058). For *post hoc* comparisons we took the significant results of the ANOVA as a substitute for an *a priori* hypothesis. That is, we are not aiming at a set of *post hoc* tests with a joined family wise error rate, but instead, investigate the driving contrasts for the significant effect already demonstrated by the ANOVA. Thus we use the Least Significant Difference (LSD), which revealed a significant effect between relative orientation task and pointing task (*p* = 0.001) but no effect between absolute orientation and relative orientation task (*p* = 0.176) or absolute orientation and pointing task (*p* = 0.066). Applying a Bonferroni correction did not change the pattern of results (relative orientation/pointing *p* = 0.003, absolute orientation/relative orientation *p* = 0.528, absolute orientation/pointing *p* = 0.198). In line with our hypothesis, we found also after 90 min of exploration a significantly better accuracy for the infinite time condition. With increased exploration time, we found as well a significant main effect for task that was mainly driven by a significantly better accuracy in the relative orientation than in the pointing task, contrary to our hypothesis.

To compare whether, as we expected, we have an effect of familiarity, we compared the mean accuracy over time and task conditions after 30 min (one session) and 90 min (three sessions) exploration of the 22 participants, who performed the exploration and test phase repeatedly. The mean accuracy over time and task conditions was after the first session 50.7% and after the third session 57.1%. We then performed a one-way repeated measure ANOVA with accuracy as dependent and session as an independent factor. We found a significant main effect for session (F(1,21) = 11.015, *p* = 0.003, η^2^ = 0.344). Supporting our hypothesis, this indicated that the task accuracy improved with the increasing familiarity of the city when explored with a map.

#### Accuracy as a Function of Clicks on Houses

**FIGURE 7:**
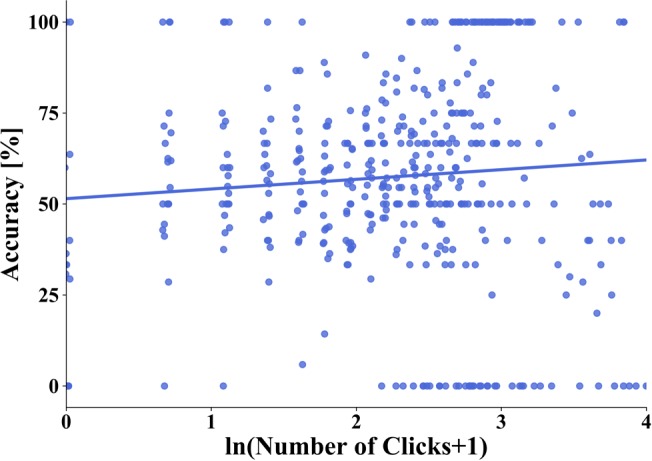
Linear regression between overall task accuracy and the natural logarithm of the number of (clicks+1) on a house after 90 min of exploration. One dot represents the average accuracy of all trials from one subject with a specific number of clicks on a house. The blue line depicts the regression line.

We hypothesized that the more frequently a house was clicked on and thus viewed during the city exploration with the interactive map, the better the task accuracy would be. For the relative orientation task, we compared the clicks on the prime and the correct target house and for the pointing task the clicks on the prime and the target house in each trial. We then used the accuracy of the house with the lower click numbers as the relevant indicator for the respective trial. With this, we calculated for each participant the accuracy averaged over the trials containing houses with none, one, and two, and so on up to the maximum of clicks observed. Because of the right-skewed distribution we applied the natural logarithm to our data. With these, we then performed a weighted linear regression averaged only over the three tasks in the infinite time condition to make results after one and three sessions comparable. The weights are equal to the number of trials contributing to the mean accuracy for each number of clicks of each participant. After 30 min exploration the weighted linear regression revealed no significant effect for number of clicks (F(1,677) = 1.336, *p* = 0.248, *R*^2^ = 0.002). This changed after 90 min of exploration. The results revealed a correlation with a slope of 0.027 and an intercept of 0.51. Statistically this relation between logarithmic (ln) number of clicks and performance revealed a significant positive effect for number of clicks (F(1,474) = 6.526, *p* = 0.011, *R*^2^ = 0.014) (Figure 7). This indicates that the accuracy improves on average by 2.7% when the number of (clicks+1) on a house increases by a factor of *e* (=2.718). In conclusion, we found a significant positive linear correlation between the number of clicks on houses and task accuracy. This supports our hypothesis that participants’ task accuracy improved the more frequently a house was viewed.

#### Accuracy as a Function of Distance

**FIGURE 8:**
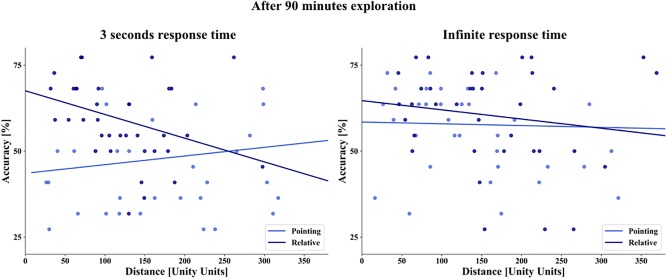
Linear regression of distance (abscissa) and overall task accuracy (ordinate) after 90 min of exploration. The blue dots depict the combination of task and house averaged over subjects, dark blue for the relative orientation task, and light blue for the pointing task on the left with 3 s response time and on the right with infinite time to respond. The straight lines depict the linear regression.

Participants learned the spatial dependencies of the city with an interactive city map. Based on previous studies, we hypothesized that we would find no influence of the distance between the houses on the task accuracy (Loomis et al., 1993; Meilinger et al., 2015). To estimate the influence of distance, we decided to only calculate the distance between two houses and not consider the complex triangular design in the relative task to perform comparable analysis to the pointing task. Therefore, we calculated the distance between prime and target house in the pointing task and between the prime and the correct target house in the relative orientation task. We investigated the distance up to 310 Unity^®^ units in the relative task and up to 371 Unity^®^ units in the pointing task representing the maximal distance of houses used in the particular task. We performed a linear regression analysis and found after one session, indeed no significant correlation between accuracy and distance in the infinite time condition for the relative orientation task (F(1,34) = 0.475, *p* = 0.495, *R*^2^ = 0.014) and the pointing task (F(1,34) = 0.236, *p* = 0.630, *R*^2^ = 0.007) (Figure 8). In line with these results, we found after three sessions in the infinite time condition no significant correlation between accuracy and distance for the relative orientation task (F(1,34) = 0.879, *p* = 0.354, *R*^2^ = 0.025) and the pointing task (F(1,34) = 0.039, *p* = 0.845, *R*^2^ = 0.001). In the 3 s response condition, we found after 90 min exploration an unexpected significant negative correlation between accuracy and distance for the relative orientation task (F(1,34) = 4.769, *p* = 0.035, *R*^2^ = 0.123). The correlation in the pointing task was not significant in this condition (F(1,34) = 0.751, *p* = 0.392, *R*^2^ = 0.021) (Figure 8). Consistent with map learning, we found no distance effect when participants learned the city layout with the interactive map with time for cognitive reasoning. Unexpectedly, with spontaneous response participants performed better with a smaller distance between stimuli when relative orientation between houses was tested.

#### Accuracy as a Function of Angular Difference

**FIGURE 9:**
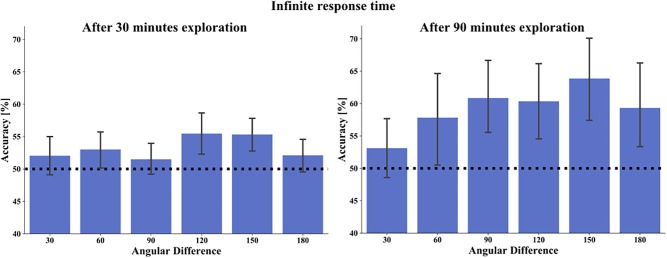
Overall task accuracy in relation to the angular difference between choices in the task stimuli, on the left after 30 min exploration and on the right after 90 min exploration. Bars depict mean accuracy in respect to 30°, 60°, 90°, 120°, 150°, and 180° categories. Error bars represent 95% confidence interval. The black dashed line marks the chance level of 50%.

Next, we hypothesized that after map learning the accuracy in the spatial tasks would depend on the size of the angular difference between stimuli choices in our 2 AFC task design. This is based on the reasoning that metric representations should make larger angular differences between the choices easier to discriminate and thus to yield better accuracy. In all tasks, participants had to compare two alternative choices that differed from each other in varying angular degrees in steps of 30°. The analysis combines deviations resulting from clockwise or anti-clockwise rotations, i.e., matching bins from 0°–180° and 180°–360° deviations are collapsed. We calculated the angular difference in the absolute orientation task between two arrows indicating cardinal directions on the stimuli and in the pointing task between two arrows pointing from a target house to a prime house. In the relative orientation task, we computed the angular difference between the orientations of two target houses. Due to the variations in the orientation of the houses, the angular difference between these angles varied from a minimum of 30° in steps of 30° with a maximal deviation of ± 5° in each step. After one exploratory session, we observed the best accuracy averaged over tasks in the infinite time condition at an angular difference between the options of 120° with 55% and slightly lower at 150°. The accuracy of all other angles was between 52 and 53%. For statistical testing, we performed a one-way repeated measure ANOVA with accuracy as the dependent variable and angular difference categories (30°, 60°, 90°, 120°, 150°, 180°) as the repeated factor. However, in spite of the large number of subjects, we could not demonstrate a significant main effect for angular difference after 30 min exploration (F(5,345) = 1.5027, *p* = 0.1883) (Figure 9). As the accuracy in the 3 s task conditions increased after three training sessions and revealed an accuracy level above chance, we included the analysis of angular differences averaged over tasks in the 3-s time condition. Here, the accuracy level fluctuated between 49% at 150° and 58% at 180°. We found no significant effect for angular difference in this condition (F(5,105) = 1.6678, *p* = 0.1488). After three exploration sessions and 90 min exploration time the overall accuracy averaged over tasks in the infinite time condition increased from the lowest level at 30° of 53.1% to the highest accuracy level at 150° with 63.9% (mean accuracy for 30° = 53.1%, 60° = 57.8%, 90° = 60.9%, 120° = 60.4%, 150° = 63.9%, 180° = 59.3%). Here, the one-way repeated measure ANOVA revealed a significant effect for angular difference (F(5,105) = 2.891, *p* = 0.017, η^2^ = 0.344) (Figure 9). *Post hoc* comparisons with LSD correction revealed a significant angular difference between 30° and 90° (*p* = 0.010), between 30° and 120° (*p* = 0.030), 30° and 150° (*p* = 0.000), and 30° and 180° (*p* = 0.043). All other comparisons were not significant (*p* between 0.098 and 0.882). Applying Bonferroni correction left the 30°/150° comparison significant (*p* = 0.007). In contrast to 30 min of exploration, we found after 90 min of exploration with the interactive city map and infinite time to respond a significant effect of angular difference on accuracy with an improved accuracy with greater angular differences.

#### Accuracy as a Function of Alignment

**FIGURE 10:**
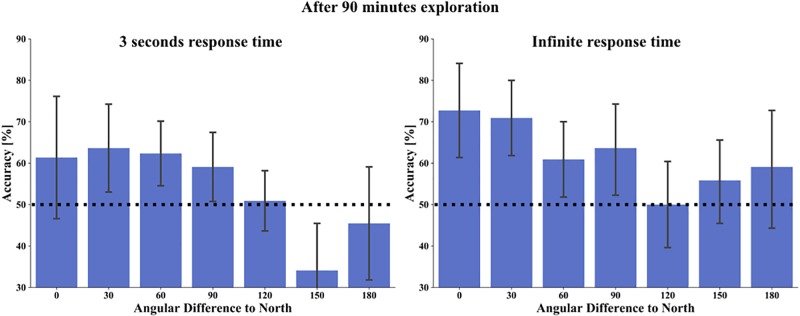
Task accuracy in the absolute orientation task in relation to an angular difference of map north to tested stimuli orientation (alignment) after 90 min exploration, on the left with 3 s response time and on the right with infinite time to respond. Bars depict mean accuracy in respect to 0°, 30°, 60°, 90°, 120°, 150°, and 180° categories. Error bars depict 95% confidence interval. The black dashed line marks the chance level of 50%.

As it is well known that the alignment of a reference direction and tested orientation improves performance accuracy or reaction time, we hypothesized that tasks’ accuracy would be better with aligned than misaligned orientation to cardinal north learned from the map. We therefore investigated the influence of angular difference of the facing direction of the correct stimulus choice in the absolute orientation task toward north on the map (Figure 10). Here, participants had only one house to consider. As before, the analysis combined deviations resulting from clockwise or anti-clockwise rotations, i.e., matching bins from 0°–180° and 180°–360° deviations were collapsed. After 90 min exploration in the 3 s response time condition we found the best accuracy with an angular difference of 30° (mean accuracy for 0° = 61.4%, 30° = 63.6%, 60° = 62.3%, 90° = 59.1%, 120° = 50.9%, 150° = 34.1%, 180° = 45.5%) and in the infinite response time condition with an aligned orientation of task stimulus to map north (mean accuracy for 0° = 72.7%, 30° = 70.9%, 60° = 60.9%, 90° = 63.6%, 120° = 50.0%, 150° = 55.8%, 180° = 59.1%). A one-way repeated measure ANOVA revealed in both time conditions a significant main effect for angular difference toward north (3 s response time: F(6,126) = 4.3315, *p* = 0.001, η^2^ = 0.171 and infinite response time: F(6,126) = 2.521, *p* = 0.024, η^2^ = 0.107). *Post hoc* comparisons revealed for the 3 s condition with LSD correction a significant accuracy difference between 0° (*p* = 0.008), 30° (*p* = 0.001), 60° (*p* = 0.000), 90° (*p* = 0.000), and 120° (*p* = 0.009) to 150° angular difference. Additionally 30°and 60° were significantly different to 180° (*p* = 0.025 and *p* = 0.038, respectively). All other comparisons revealed no significant angular differences (*p* between 0.054 and 0.911). Applying Bonferroni correction resulted in a significant difference of 150°–30° (*p* = 0.021), to 60° (*p* = 0.009), and to 90° (*p* = 0.010). The results revealed here a significantly worse accuracy, when the correct stimulus choice deviated by 150° to the map north. *Post hoc* comparisons for the infinite time condition revealed with LSD correction a significant angular difference between 0° and 120° (*p* = 0.004), and 0° and 150° (*p* = 0.050). Furthermore, accuracy for 30° (*p* = 0.021), 60° (*p* = 0.030), and 90° (*p* = 0.043) were significantly different from 120° angular difference. All other comparisons revealed no significant accuracy differences (*p* between 0.07 and 0.837). Applying Bonferroni correction resulted in no significant pairwise difference. In summary, for both time conditions we found the best accuracy when the correct stimulus choice and map north were aligned or nearly aligned, with a significant dependence of accuracy on the angular difference.

#### Accuracy as a Function of FRS Scaling

**FIGURE 11:**
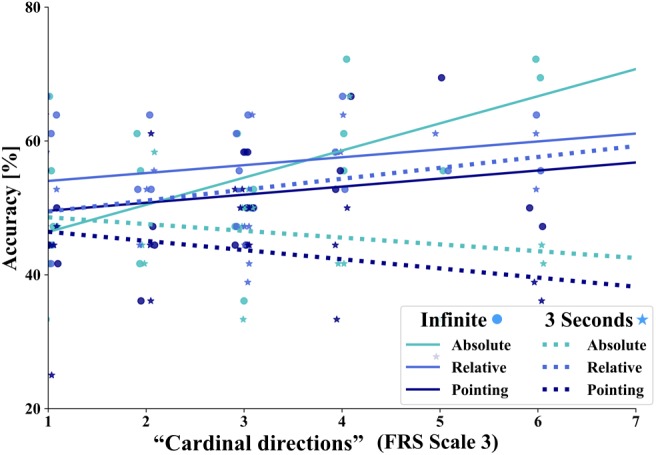
Linear regression between task accuracy (*y*-axis) and scale 3 “cardinal directions” of the FRS questionnaire (*x*-axis). The straight lines depict the correlations: turquoise line for the absolute orientation task, middle blue line for the relative orientation task and dark blue line for the pointing task. Solid lines depict tasks in the infinite time condition and dashed lines in the 3 s response condition, respectively. The dots represent the single data points of the tasks in the respective colors for the infinite response condition and the stars for the 3 s time condition.

To estimate subjectively rated abilities in spatial orientation strategies and their influence on task accuracy, we performed the FRS questionnaire (Münzer and Hölscher, 2011; Münzer et al., 2016b). This questionnaire consists of three scales comprised of Likert items with a score ranging from 1 to 7 (1 = strongly disagree to 7 = strongly agree). Scale 1, the “global-egocentric orientation” scale captures general ability and egocentric strategies based on knowledge of routes and directions. Scale 2, the “survey” scale evaluates an allocentric strategy for mental map formation. Scale 3, the “cardinal directions” scale measures the knowledge of cardinal directions. We calculated the Pearson correlation between the three FRS scales for our subjects (scale 1: *M* = 4.442, SD = 1.2; scale 2: *M* = 4.011, SD = 1.392; scale 3: *M* = 2.875, SD = 1.446). We found a strong positive significant pairwise correlation between all three scales of the FRS questionnaire (scale 1/scale 2: *r* = 0.577, *p* < 0.001; scale 1/scale 3: *r* = 0.418, *p* = 0.001; scale 2/scale 3: *r* = 0.528, *p* = 0.000) in accordance with previous research (Münzer and Hölscher, 2011). We then calculated linear regressions between the FRS scales and tasks accuracies after three exploration sessions (Figure 11). We investigated, whether ratings of the three spatial strategy scales that captures strategy use learned in real environments, would have an impact on spatial learning with the interactive map of a city. We found no significant correlations for scale 1 and 2 with tasks accuracies (Scale 1: absolute/3 s – *R*^2^ = 0.105, *p* = 0.140, absolute/inf – *R*^2^ = 0.069, *p* = 0.234, relative/3 s – *R*^2^ = 0.166, *p* = 0.059, relative/inf – *R*^2^ = 0.030, *p* = 0.436, pointing/3 s – *R*^2^ = 0.023, *p* = 0.495, pointing/inf – *R*^2^ = 0.171, *p* = 0.055) (Scale 2: absolute/3 s – *R*^2^ = 0.120, *p* = 0.113, absolute/inf – *R*^2^ = 0.160, *p* = 0.064, relative/3 s – *R*^2^ = 0.166, *p* = 0.059, relative/inf – *R*^2^ = 0.043, *p* = 0.353, pointing/3 s – *R*^2^ = 0.095, *p* = 0.161, pointing/inf – *R*^2^ = 0.063, *p* = 0.258). Interestingly, for the scale “cardinal directions” (scale 3) we found a significant correlation with the accuracy in the absolute orientation task in the infinite time condition (F(1/20) = 10.629, *p* = 0.003, *R*^2^ = 0.347). All other correlations were also for scale 3 not significant (Scale 3: absolute/3 s – *R*^2^ = 0.037, *p* = 0.385, relative/3 s – *R*^2^ = 0.123, *p* = 0.108, relative/inf – *R*^2^ = 0.057, *p* = 0.280, pointing/3 s – *R*^2^ = 0.041, *p* = 0.363, pointing/inf – *R*^2^ = 0.056, *p* = 0.287). Our finding suggests that participants who rate themselves as knowing cardinal directions in real environments well also perform with better accuracy when judging houses orientations toward north after exploration of a virtual city with an interactive map.

## Discussion

In the present study, we investigated the learning of spatial properties after exploration of a large-scale virtual city with an interactive city map after one and three 30-min sessions. After each session, we performed three tasks adapted from a previous study (König et al., 2017) to measure spatial knowledge of the orientation of houses’ facing directions toward cardinal north (absolute orientation), orientation of the facing directions of houses in relation to the orientation of a prime houses facing direction (relative orientation), and the location of two houses to each other (pointing task). Our results revealed that one-time exploration with the interactive city map was sufficient to explore most of the city, but not enough to acquire spontaneous spatial knowledge, which improved with time for cognitive reasoning in line with our hypothesis. After three exploration sessions, the overall task accuracy increased significantly revealing a familiarity effect. With an increased accuracy level, we found a main effect for task that was mainly driven by a better accuracy in the relative orientation than in the pointing task. Spatial knowledge retrieval was still more accurate with the unrestricted time to respond. The familiarity effect of increased exploration time was accompanied by an increased accuracy the more often a house was viewed. With better task accuracy after 90 min of exploration, we found an angular difference effect between task choices with an improved accuracy with greater angular differences. Consistent with map learning, we found an alignment effect with the best accuracy when the correct stimulus choice and map north were closely aligned. As hypothesized, our results revealed no distance effect when participants learned the city layout with the interactive map with time for cognitive reasoning. Unexpectedly though, with a spontaneous response, task accuracy was better with a smaller distance between stimuli when relative orientation between houses was tested. Evaluating the abilities of self-reported spatial orientation strategies learned in real environments with the FRS questionnaire, revealed a positive correlation between participants’ rates on the scale “cardinal directions” onto judging houses orientations toward cardinal north after map exploration. We argue that these results suggest that after interactive map exploration of a virtual city information is memorized with respect to the metric information that is available from the map.

In the present study, there are some points to be considered. Firstly, we observed after 30 min of exploration with the interactive city map and unlimited time for cognitive reasoning in the retrieval tasks an accuracy level only slightly above chance. Our experimental design is in line with previous studies in which participants also performed 20–30 min training with the environment and a subsequent test phase (Shelton and McNamara, 2001; Mou and McNamara, 2002; McNamara et al., 2003; Greenauer and Waller, 2008; Mou et al., 2009; Marchette et al., 2011). Nevertheless, in relation to previous studies the extent and complexity of our virtual city were considerable. Additionally, the spatial task design in the current study was based on the front on screenshots of houses taken in our virtual city from a pedestrian perspective to replicate the study of König et al. (2017) in a virtual city. Therefore, learning the city layout with a city map required an interactive feature that combined the map with the front on views of the houses. This lead to a necessary change of perspective, when participants viewed the map from a bird’s eye perspective to watching the house fronts from a pedestrian perspective. Previous research found that recalling spatial information in a different orientation or perspective than it was learned and stored, yields costs in terms of increased errors or delays (Shelton and McNamara, 2004; McNamara et al., 2008; Meilinger et al., 2013). We, therefore, propose that the perspective switch in our task design added to the difficulty of the design.

Furthermore, performing the experiment in a laboratory environment, it is obvious to the participants that they are tested in some respect. Based on the experience of a separate pilot study, we wanted to avoid in the main study that participants focus on irrelevant aspects like the ornaments of houses, but to ensure that all participants were aware of required spatial learning. We therefore introduced our tasks before the exploration phase including example trials of all task and time conditions. In this way all participants were able to intentionally pay attention to relevant spatial features while freely exploring the city with the map. In addition, when performing several sessions, introducing the spatial tasks before the first exploration session helps to make the comparison of first and later sessions valid. Even though Burte and Montello (2017) found no learning effect of intentionality on spatial knowledge acquisition, we have to consider an effect onto our results.

In the present study, we used two conditions with 3 s and infinite time allowed for a response. These can be understood on the basis of Dual-Process Theories (Evans, 1984, 2008; Finuncane et al., 2000; Kahneman et al., 2002). Response decisions within 3 s require rapid and intuitive cognitive processes in line with “System 1” processes. On the other hand, infinite time to respond allows for time consuming slow, deductive and analytic “System 2” processes. After one and still three exploration sessions, accuracy was better with unrestricted time for cognitive reasoning, which is in line with Kahneman et al. (2002) who argued that “System 1” processes improve with cognitive processes descending from “System 2” processes but need increased proficiency. Thus, our results indicate that all subjects were still in an early phase of spatial learning and that cognitive reasoning contributed heavily to accuracy.

Another important factor is the exploration time with the map. Getting to know a real-world environment takes place over an extended period. In contrast to the classical framework of spatial knowledge acquisition (Siegel and White, 1975), which proposes a stepwise qualitative switch in spatial learning with increased familiarity, the framework proposed by Montello (1998) suggests that increased familiarity with the environment leads to more accurate and complete spatial knowledge in a more quantitative nature that might improve over years and even decades. The latter was supported by empirical studies that repeatedly showed that increased familiarity with an environment improves accuracy, extend, and reliability of spatial knowledge (Thorndyke and Hayes-Roth, 1982; Lindberg and Gärling, 1983; Montello, 1998; Ishikawa and Montello, 2006; Burte and Montello, 2017). In line with these findings, the exploration behavior revealed that one 30’ session was sufficient to look at the vast majority of houses on the map of the city and most of the houses were viewed even several times. After 90 min of map exploration of the city, nearly all participants explored the whole city layout and viewed all houses repeatedly. The longer exploration time lead to a significant increase in task accuracy, thus revealing a positive familiarity effect with the city layout that is compatible with the proposal by Montello (1998). Looking at a house more often might reflect that participants found specific houses more difficult to memorize and, therefore, clicked more often onto these houses. Nevertheless, the results revealed higher task accuracy with an increase in how often a house was viewed. Concluding, our exploration and task design is demanding, but increased time to explore the virtual city with the interactive map enhances familiarity with the city layout and improves knowledge of spatial properties observable in increased task accuracy.

In the current study, we investigated adapted spatial tasks of a previous report (König et al., 2017) in a highly controlled laboratory setup, with the possibility to differentially investigate spatial learning with either a map or direct experience. That previous publication studied spatial knowledge after at least one-year experience in the hometown of Osnabrück. Spatial learning in that situation combines different sources for learning, e.g., direct experience and maps (Richardson et al., 1999; Ishikawa and Montello, 2006; Meilinger et al., 2013). König et al. (2017) reported a better knowledge of relative orientation between two houses than of absolute orientation of one house toward cardinal north with a spontaneous decision and a reversal of that relation with time for cognitive reasoning. In that paper, with a spontaneous decision, the best performance was found in pointing from one house to another house. Those results were argued to be in line with an action-oriented embodied approach to spatial cognition (Engel et al., 2013). In the present study, analyzing the task accuracy after exploration with an interactive map of our virtual city revealed that after 90 min exploration the best performance was found in the relative orientation task with significantly better accuracy than in the pointing task. In contrast to the previous study in which task accuracy was best in the pointing task, in the present study accuracy in the pointing task was lowest after 30 min map exploration in the 3 s condition as well as after 90 min map exploration with 3 s and infinite response time. Taken together, learning with an interactive city map suggests supporting the learning of spatial properties that use orientation information in relation to cardinal north or relative orientation between two houses, whereas spatial properties acquired by living in a city enhanced preferentially the ability for straight line pointing between two houses.

However, a comparison of learning by direct experience in the identical environment of Seahaven is desirable. This can be performed in the virtual environment and even combined with further measurement techniques like eye tracking (Clay et al., 2019). When learning in the virtual environment, we would expect that the pointing task achieves the highest task accuracy, followed by the relative and absolute orientation tasks. Furthermore, with direct experience we expect a reduction or absence of the alignment effect, as the spatial knowledge might not be acquired in a specifically oriented reference frame. Finally, the correlation of task accuracy to the scales of the FRS questionnaire, which reflects spatial strategies learned in real environment, should be enhanced. In summary, a comparison of map learning with learning by direct experience in Seahaven will bear the full potential of the study design.

Investigating the effect of angular differences between tested stimuli choices, the previous study found an angular difference effect indicated by a better performance with larger angular differences only in tasks that had performance levels above 55%. After a single session of spatial learning with our interactive city map, all task accuracy levels were below 55%. Thus, the lack of angular difference effect is not surprising. Though, improved task accuracy after 90 min of city exploration with the map revealed in the current study a significant angular difference effect with a positive correlation between task accuracy and larger angular differences between stimuli choices.

Getting to know a new environment with the aid of a map is supposed to support the acquisition of survey knowledge (Thorndyke and Hayes-Roth, 1982; Taylor et al., 1999; Frankenstein et al., 2012; Meilinger et al., 2013, 2015). Survey knowledge acquired from maps includes topographical properties of the learned environment, e.g., the locations of objects relative to a coordinate system and inter-object distances and direction information (Thorndyke and Hayes-Roth, 1982; Richardson et al., 1999; Montello et al., 2004) This also suggests that this knowledge is coded in an allocentric reference frame (Richardson et al., 1999; Montello et al., 2004). Thorndyke and Hayes-Roth (1982) found that learning with a map leads to survey knowledge, which enabled people to estimate straight-line distances. In that study, a pointing task, which is often understood as a measure of survey knowledge (Montello et al., 2004), performed in the real environment after map learning disclosed more errors in pointing to unseen objects. In the present study, in line with Thorndyke and Hayes-Roth (1982), pointing from one house to another was less accurate than learning of orientation information. Even though it was not required or suggested that participants egocentrically orientate themselves in our task design, we cannot rule out that some tried to do so. In this case, primarily restricted response time would lead to misorienting as discussed by previous research (Thorndyke and Hayes-Roth, 1982; Montello et al., 2004) and cause an overall lower accuracy. Comparing learning with a 2D and a 3D city map, researchers found better performance in spatial tasks after learning with a 2D map, even with participants who were experienced in virtual reality games (Oulasvirta et al., 2009). In line with this study, we have to consider that the interactive map was harder to learn. It might especially be more difficult to integrate memorized knowledge to be able to perform the pointing task. This is in line with previous research that suggested that it is more demanding to integrate spatial knowledge to form survey knowledge (e.g., Golledge et al., 1993; Montello, 1998). In summary, pointing from one house to another, understood, as a task to measure survey knowledge, was less accurate as knowledge of houses orientation toward north or relative orientation to another house based on metric information of the map.

Reference frames are essential to organize learning and memory of spatial properties in small and large environments (Greenauer and Waller, 2010). For actual navigation in the environment, allocentric spatial relations are recalled and are combined with present egocentric information (Mou et al., 2004; Riecke et al., 2007; Kelly and McNamara, 2008). Beside egocentric and allocentric reference frames intrinsic object-to-object relation is an important reference to learn and memorize spatial relations (Mou and McNamara, 2002). Egocentric spatial cues, which are combined with allocentric environmental cues, are supposed to be the primary cues for building an intrinsic reference frame (McNamara, 2002). Spatial information about cardinal directions and orientations are given by a map and thus directly provide spatial information in an allocentric reference frame. In our study, the setup of the map training makes use of an allocentric reference frame related to absolute north intuitive as our interactive map depicts a conventional two-dimensional city map of our virtual city with north being up on the map. In line with this, we found with time for cognitive reasoning that participants learned knowledge of the cardinal orientation of houses and relative orientations between two houses nearly equally well after three exploration sessions. In the spontaneous response condition increased exploration time thus increased familiarity was needed to perform above chance level. After 90 min exploration we found an improved accuracy with spontaneous response for the absolute orientation toward north as well as the relative orientation between two houses but with better accuracy for the latter. Our results suggest that allocentric spatial information provided by the map is learned and leads to knowledge of houses orientations to cardinal directions and relative house orientations to each other. Spontaneous knowledge retrieval needs a better familiarity achieved by an increased exploration time.

Learning a spatial layout of a large-scale environment with a map provides the information with respect to an allocentric reference frame in the form of cardinal directions. Having a fixed reference frame is also supposed to lead to the acquisition of a global reference frame (Richardson et al., 1999; Frankenstein et al., 2012; Meilinger et al., 2015). Survey tasks can also be solved using global reference frames that are learned from experience leading to a decrease in performance with greater distance between tested objects (distance effect) (Loomis et al., 1993; Meilinger et al., 2015). In contrast, using a global reference derived from a map is supposed to yield no distance effect (Frankenstein et al., 2012), because a map directly provides an overview giving distances and locations as well as spatial orientation toward cardinal directions in proportion to the depicted environment. In line with previous research in the presented study, we did not find a distance effect on performance in our tasks after learning with our interactive map with time for cognitive reasoning. Unexpectedly, testing the relative orientation between two houses with spontaneous response smaller distances between tested houses were positively correlated with task accuracy. A speculative interpretation is that at that time in exploration local snapshots were not yet merged into a global reference frame, giving rise to a distance effect. However, this observation awaits independent confirmation. Otherwise, the absence of a distance effect in most tasks suggests that participants acquired the knowledge of a global reference frame with our interactive city map.

Another spatial property that is learned when using a map with a single preferred orientation, like cardinal north is up on a map, is the orientation specificity of remembered spatial knowledge (Thorndyke and Hayes-Roth, 1982; Presson and Hazelrigg, 1984; Montello et al., 2004). When spatial knowledge is retained orientation specific, participants perform best when remembered, and tested orientations are aligned. Mou and McNamara (2002) found that objects’ locations were remembered in relation to a salient intrinsic reference frame defined by the layout of the environment even when this was not aligned to the egocentric experience. Testing pointing accuracy in a large-scale environment that was learned by active navigation, McNamara et al. (2003) found an improved pointing accuracy when tested objects were aligned with salient aspects of the environment, which serve as the reference in an allocentric frame. Also, Brunyé et al. (2015) found that, when the environment was explored by active navigation, cognitive maps can preferably be oriented toward salient environmental features, thus in allocentric terms. In cases where the salient environmental features were aligned with north, pointing tasks revealed the best performance when these features were aligned to the north direction (Marchette et al., 2011; Frankenstein et al., 2012; Brunyé et al., 2015). Previous research suggested as the main factor for orientation specificity of spatial knowledge the source of spatial learning (Presson and Hazelrigg, 1984). Spatial knowledge acquisition by direct sources like walking or viewing an environment revealed no alignment whereas learning with indirect sources like a map led to consistent alignment effects. Similar to the relative heading task of Burte and Hegarty (2014), we investigated the orientation of photographs facing direction toward cardinal north and relative orientation of facing directions of two photographs in respect to a prime reference house. In line with previous research (Evans and Pezdek, 1980; Levine et al., 1984; Presson and Hazelrigg, 1984; MacEachren, 1992; Montello et al., 2004; Shelton and McNamara, 2004), we found an alignment effect between north up map orientation and tested house orientation. This suggests that spatial knowledge was acquired with a specific orientation and thus supports previous findings.

Though, previous research found large individual differences in spatial knowledge acquisition (Montello, 1998; Hegarty et al., 2002, 2018; Ishikawa and Montello, 2006; Wolbers and Hegarty, 2010; Burte and Montello, 2017). For example, Ishikawa and Montello (2006) found substantial differences in learning survey knowledge. Evaluations of self-reports on spatial abilities were astonishingly good to predict spatial task performances. The frequently used Santa Barbara scale of Directions (SBSOD) (Hegarty et al., 2002) measures a self-reported SOD, which captures the ability to orient and navigate while moving in an environment. The SBSOD scale primarily evaluates subjectively rated differences in navigation proficiency (Montello, 2018), and was positively correlated to a variety of skills. This includes individual differences in survey knowledge (Hegarty et al., 2002; Burte and Montello, 2017), in pointing to locations in a known environment (Sholl, 1988; Hegarty et al., 2002), of spatial knowledge in a newly learned environment (Hegarty et al., 2006), of judging own facing directions in relation to landmarks facing directions in the allocentric heading-recall task (Sholl et al., 2006; Burte and Hegarty, 2012), and recently also to the ability to point to north (Brunyé et al., 2015; Burte and Montello, 2017). As indicated by Wolbers and Hegarty (2010) being a successful navigator might be chiefly determined by choosing the right spatial strategy for a spatial task. In our study, we, therefore, wanted to evaluate whether subjective reports of spatial orientation strategies evaluating spatial knowledge based on egocentric or allocentric reference frames learned in real environments would also predict learning of spatial properties with a map of a virtual city. For this purpose, we used the “Fragebogen Räumliche Strategien” FRS (translated: Questionnaire of Spatial Strategies) (Münzer and Hölscher, 2011), which measures spatial orientation strategies on three scales in respect to an egocentric reference frame, allocentric reference frames and cardinal directions explicitly. Further, it was validated to predict spatial learning in real environments. Comparing the scales’ ratings with our tasks accuracies, we found a positive correlation between the scale “cardinal directions” and the absolute orientation task, which measures participants’ knowledge of houses orientations toward the north cardinal direction with the unrestricted response time. In conclusion, comparing self-reported egocentric and allocentric spatial orientation strategies with task accuracy in our experiment revealed a positive correlation between allocentric knowledge of cardinal directions learned in real environments and after exploration of a virtual city with a map.

## Conclusion

In the present study, we investigated the learning of spatial properties after exploration of a large-scale virtual city with an interactive city map in a controlled setup. Our results revealed that our design was valid with most of the participants viewing nearly all of the houses a few times after three exploration sessions. Further, with more familiarity after increased exploration time task accuracy improved and revealed task differences. These were mainly caused by a better accuracy in judging the relative orientation of houses facing directions than in straight line pointing between two houses. In line with previous research on spatial learning with a map, our results further suggest that participants acquired a global reference frame when learning with the map indicated by a lack of distance effect. We assume that acquired spatial knowledge was orientation specific, which was revealed by an alignment effect between learned map north orientation and the orientations of the facing directions of the tested house. These results indicate that solely learning with a map, spatial information is acquired and memorized based on orientation information directly available from the map. The next step in future research is to investigate the learning of spatial properties while exploring the virtual city in VR. This offers the possibility of comparing acquired spatial knowledge with controlled learning either with the interactive city map or directly navigating in VR, giving insight into differently learned spatial properties.

## Ethics Statement

This study was approved by the Ethics Committee of the Osnabrück University in accordance with the ethical standards of the Institutional and National Research Committees.

## Author Contributions

SK designed, supervised, and managed the study, analyzed the data, wrote, and revised the manuscript. VC built the virtual city, wrote the scripts, analyzed the data, and revised the manuscript. DN built the map, wrote the code for the map, and revised the manuscript. LD measured the participants, wrote the scripts, and analyzed the data. NK measured the participants, wrote code for task setup, and revised the manuscript. PK designed and supervised the study, and revised the manuscript.

## Conflict of Interest Statement

The authors declare that the research was conducted in the absence of any commercial or financial relationships that could be construed as a potential conflict of interest.
